# Radiocapitellar Alignment in Suspected Pediatric Monteggia Lesions: Narrative Review and Imaging Interpretation Framework

**DOI:** 10.3390/diagnostics16132105

**Published:** 2026-07-05

**Authors:** Xiaoyue Li, Fei Gao, Jingmiao Wang, Baisong Chen, Taichun Li

**Affiliations:** 1 Department of Pediatric Surgery, Huzhou Maternal and Child Health Care Hospital, Huzhou 313000, China; lxyped@163.com; 2 Department of Pediatric Orthopedics, Shanghai Children’s Hospital, Shanghai 200062, China; gaofei@shchildren.com.cn (F.G.); wangjingmiao@shchildren.com.cn (J.W.)

**Keywords:** Monteggia lesion, Monteggia fracture-dislocation, radiocapitellar line, pediatric elbow, pediatric radiography, ulnar bow sign, ultrasound, arthrography, imaging interpretation framework

## Abstract

Pediatric Monteggia fracture-dislocations may be overlooked when ulnar injury is incomplete or plastically deformed and radiocapitellar malalignment is subtle. Radiographic interpretation is further complicated by incomplete ossification, non-standard projection, and the limitations of applying a single alignment line across all ages and imaging scenarios. This narrative review synthesizes clinically relevant evidence on radiocapitellar alignment assessment in suspected pediatric Monteggia lesions and proposes an imaging interpretation framework for radiographs and problem-solving imaging. The review integrates developmental anatomy, radiographic adequacy, radiocapitellar line behavior, forearm-based P-line assessment, lateral humeral line assessment, ulnar bow sign, and targeted second-line imaging with ultrasound, MRI, and arthrography. The framework emphasizes three practical steps: first determining whether the available images are adequate for line-based assessment; then selecting the line or sign according to age, ossification stage, projection, and available landmarks; and finally reporting discordant or limited studies as equivocal rather than forcing a binary normal/abnormal interpretation. We also summarize diagnostic error considerations, structured reporting elements, and future directions for AI-assisted measurement and uncertainty-aware interpretation. The proposed framework is intended to support consistent radiologic reasoning and communication among emergency physicians, radiologists, and pediatric orthopedic surgeons. It is not a validated diagnostic rule, and prospective observer-performance studies are needed before implementation as a clinical pathway.

## 1. Introduction

Pediatric Monteggia lesions are classically defined as ulnar fracture or deformity associated with dislocation or subluxation of the radial head. Since Bado’s original classification, the lesion has been understood as an ulna–radial head injury unit rather than an isolated fracture or isolated radial head dislocation [1]. In children, recognition remains challenging because the ulnar injury may be incomplete, minimally displaced, or plastically deformed, and the radiocapitellar abnormality may be subtle. Classic and contemporary reviews have repeatedly emphasized that Monteggia injuries in children include a spectrum of patterns and that incomplete recognition of the ulnar component can lead to missed or delayed diagnosis [2,3,4,5].

Missed pediatric Monteggia lesions are not a new problem. Early radiology literature specifically described the missed Monteggia fracture as a diagnostic pitfall, and later reviews and observer-performance studies continued to emphasize delayed recognition, particularly when the injury is interpreted as an isolated forearm or ulnar fracture [2,3,4,6]. Modern reviews and observer-performance studies support the same concern: diagnosis depends not only on image quality, but also on whether the reader actively evaluates radiocapitellar alignment, subtle ulnar plastic deformation, and injury pattern as a whole [3,6]. Monteggia-equivalent or variant lesions further broaden the diagnostic challenge, because the ulna may show plastic deformation or atypical injury patterns rather than a complete, obvious fracture-dislocation pattern [7,8].

The radiocapitellar line (RCL) is the most commonly used radiographic screening tool, but its interpretation is age- and projection-dependent. In younger children, the ossified capitellum does not necessarily represent the center of the cartilaginous capitellum. MRI studies have shown that the RCL may align with the cartilaginous capitellum even when it appears eccentric relative to the ossified capitellar nucleus [9,10]. Radiographic studies of normal children likewise demonstrate that RCL behavior varies with age, projection, and line-drawing technique [11,12,13,14]. Therefore, a binary interpretation based only on whether the RCL crosses the middle third of the ossified capitellum may be misleading, particularly in young children and on AP radiographs.

Several complementary diagnostic tools have been proposed for specific imaging scenarios. The P-line was developed for pediatric forearm radiographs, particularly when standard elbow views are unavailable [15]. The lateral humeral line (LHL) provides an AP/coronal-plane adjunct, especially when lateral displacement is suspected [16]. The ulnar bow sign helps identify plastic deformation of the ulna, a key clue in occult Monteggia-equivalent injuries [17,18]. When radiographs remain equivocal, ultrasound, MRI, and arthrography may clarify cartilaginous congruity, soft tissue interposition, or occult subluxation [19,20,21].

This review is intended for radiologists, emergency physicians, and pediatric orthopedic surgeons who interpret initial or problem-solving imaging in children and adolescents with elbow or forearm trauma. The primary setting is acute or potentially missed pediatric Monteggia injury rather than chronic surgical planning. We synthesize literature on developmental pitfalls, projection-related limitations, line-based assessment methods, ulnar morphology, targeted problem-solving imaging, and diagnostic error. The aim is to propose an imaging interpretation framework that makes the evidence chain explicit and highlights limited or discordant radiographic studies; prospective testing is required before it can be used beyond reporting and teaching support.

## 2. Literature Search and Evidence Selection

This article was designed as a targeted narrative review with a proposed imaging interpretation framework. The objective was to synthesize clinically relevant evidence on radiocapitellar alignment assessment in suspected pediatric Monteggia lesions, with emphasis on developmental anatomy, projection-related pitfalls, line-based assessment methods, ulnar plastic deformation, problem-solving imaging, diagnostic error, and emerging automated image interpretation. The review was not designed as a protocol-driven systematic review or scoping review, and quantitative pooling was not performed.

Targeted PubMed/MEDLINE searches were performed during manuscript development, and the literature set was last updated on 1 March 2026. Search concepts included combinations of Monteggia, Monteggia fracture-dislocation, pediatric or paediatric, radiocapitellar alignment, radiocapitellar line, Støren line, P-line, lateral humeral line, radiocoronoid line, ulnar bow sign, pediatric elbow ossification, ultrasound, MRI, arthrography, diagnostic error, observer performance, artificial intelligence, and automated interpretation. PubMed/MEDLINE was selected as the primary source because this review focused on peer-reviewed biomedical, pediatric orthopedic, and radiologic literature rather than gray literature or guideline development. Backward citation tracking of relevant original studies and reviews was used to capture foundational radiographic, orthopedic, and imaging studies that may not be easily identified by contemporary keyword searches. Publicly available practice resources were reviewed separately to contextualize current clinical teaching and were not counted as part of the literature evidence set.

Records were considered relevant if they contributed to one or more of the following domains: pediatric Monteggia definition and missed-injury background; elbow ossification and normal radiographic variation; radiographic quality control; RCL, P-line, LHL, radiocoronoid line, or ulnar bow sign; ultrasound, MRI, arthrography, CT, contralateral comparison, or fluoroscopic assessment for unresolved alignment questions; diagnostic error or observer performance; and automated or AI-assisted interpretation. Treatment-focused studies were retained only when they addressed consequences of delayed diagnosis, imaging pitfalls, or diagnostic context.

Candidate records identified from the targeted searches, reference chaining, and the authors’ full-text source library were screened for relevance to pediatric Monteggia recognition or radiocapitellar alignment interpretation. After de-duplication and exclusion of papers not addressing pediatric Monteggia lesions, radiocapitellar alignment, pediatric elbow ossification, line-based interpretation, problem-solving imaging, or diagnostic error, 57 literature records were retained in Supplementary Table S1. Because the review was not prospectively registered as a systematic or scoping review, a formal PRISMA flow diagram and exclusion log were not generated. Supplementary Table S1 records study design, population, imaging modality, index test or sign, reference standard or comparator where applicable, evidence directness, major findings, limitations, use in the proposed framework, and main-text citation status in the revised manuscript. The main text cites the records most directly supporting the narrative, tables, and framework; broader contextual records remain available in Supplementary Table S1.

### 2.1. Quality Safeguards for Narrative Synthesis

To reduce subjectivity inherent to narrative synthesis, study extraction and evidence categorization were performed by two authors (X.L. and F.G.) and reviewed by the senior authors (B.C. and T.L.). Evidence directness was assigned according to whether a study provided direct Monteggia diagnostic evidence, Monteggia-related clinical or missed-injury evidence, problem-solving imaging evidence, indirect normal-elbow or developmental evidence, projection or measurement-method evidence, observer-performance or diagnostic-error evidence, contextual background evidence, or AI/automation development evidence. Disagreements in categorization were resolved by discussion. The synthesis was organized around clinical imaging questions rather than by imaging modality alone.

This narrative approach was guided by SANRA-style quality safeguards for narrative reviews, including explicit scope definition, transparent literature identification, balanced citation of key evidence, and clear separation between evidence-supported statements and proposed framework elements [22]. The proposed framework should therefore be regarded as a conceptual framework for interpretation and reporting, not as a validated clinical prediction rule. The heterogeneity of the included literature, including normal-elbow developmental studies, Monteggia-specific studies, small problem-solving imaging series, and observer-performance studies, precluded diagnostic-accuracy pooling. Formal database-level PRISMA screening, risk-of-bias scoring, and a formal exclusion log were not performed; these limitations are addressed in the Section 13.

### 2.2. Use of Generative AI Tools

During manuscript preparation, ChatGPT (GPT-5.5 Pro; OpenAI, San Francisco, CA, USA) was used only for language polishing, editorial organization, and formatting assistance. The tool was not used to generate original data, perform literature selection, analyze or interpret data, or draw scientific conclusions. All content was reviewed and approved by the authors.

## 3. Existing Practice Guidance and Unmet Need

Selected publicly available practice resources consistently emphasize that pediatric Monteggia injuries are easily missed and that adequate imaging must include assessment of the radiocapitellar joint. This section is not a systematic guideline review; it is included only to clarify how the present framework relates to commonly available practice guidance. The POSNA study guide recommends AP and lateral forearm radiographs together with elbow and wrist imaging, checks radiocapitellar alignment on all views, warns that ulnar plastic deformation may be subtle, and notes that contralateral elbow radiographs or fluoroscopic/dynamic assessment may help when the diagnosis remains unclear [23]. The Royal Children’s Hospital emergency guideline similarly recommends AP and lateral forearm radiographs including the wrist and elbow, true AP and lateral elbow views for radiocapitellar assessment, and review of the posterior ulnar border for plastic deformation [24]. These resources support radiographic adequacy control before line-based interpretation.

Selected resources reviewed for this narrative article did not provide a detailed age-, ossification-, and projection-aware pathway for interpreting radiocapitellar alignment when radiographs are discordant or landmarks are incompletely ossified. In particular, general teaching that the radial shaft or radiocapitellar line should point toward the capitellum does not fully address situations in which the ossified capitellum does not represent the cartilaginous center, the AP and lateral views disagree, or a forearm-only examination lacks landmarks required for specific methods such as the P-line. The unmet need addressed here is therefore not whether radiocapitellar alignment should be checked, but how radiographic findings may be interpreted and reported when age, ossification stage, projection quality, ulnar morphology, and available views complicate that check. Table 1 summarizes how the major evidence domains link to the proposed framework.

## 4. Developmental Anatomy and Ossification-Related Pitfalls

Radiocapitellar alignment is more difficult to interpret in children than in adults because the pediatric elbow is not fully ossified on radiographs. Before skeletal maturity, a substantial part of the distal humerus and proximal radius remains cartilaginous. Plain radiographs show only the ossified portions of the capitellum, radial head, trochlea, olecranon, and epicondyles, whereas the true cartilaginous articular surfaces and their geometric centers are not directly visible [30,31]. A radiographic line drawn toward an ossified nucleus may therefore not reflect the true relationship between the radial head and the cartilaginous capitellum. This developmental discrepancy is one of the major reasons why a binary interpretation of the RCL can be misleading in young children.

### 4.1. Ossification Sequence: Why Chronological Age Alone Is Insufficient

The sequence of secondary ossification centers in the pediatric elbow is commonly remembered as CRITOE: capitellum, radial head, internal or medial epicondyle, trochlea, olecranon, and external or lateral epicondyle [31]. This sequence helps distinguish normal ossification from displaced fracture fragments. However, chronological age alone is an imperfect guide. The timing of appearance and fusion varies among children, and sex-related differences in elbow ossification have been reported, with girls generally ossifying earlier than boys [30,32,33]. For this reason, the relative order of ossification centers and the clinical context are generally more useful than a rigid age threshold.

This principle is particularly relevant to suspected Monteggia lesions. In younger children, the radial head may be incompletely ossified or not yet visible, and the ossified capitellum may represent only part of the cartilaginous capitellum. The visible ossification center is therefore not a perfect surrogate for the articular center. Other pediatric elbow lines show similar age-related limitations; for example, the relationship of the anterior humeral line to the capitellar ossific nucleus varies with age [32]. Although this finding concerns a different line, it reinforces a broader diagnostic principle: in children, line-based interpretation is most reliable when linked to ossification stage rather than reduced to a fixed adult-like rule.

### 4.2. Eccentric Capitellar Ossification and Apparent RCL Abnormality

The classical teaching that the RCL is expected to pass through the center or middle third of the capitellum assumes that the ossified capitellum represents the center of the cartilaginous capitellum. MRI studies show that this assumption is often invalid in young children. Fader et al. demonstrated that capitellar ossification is eccentric rather than concentric: the ossified capitellum is initially positioned anteriorly and proximally on the sagittal plane and medially on the coronal plane, then gradually migrates posteriorly, distally, and laterally with age [10]. This developmental pattern explains why the RCL may appear eccentric relative to the ossified capitellum even when radiocapitellar congruity is normal.

The same group compared the relationship of the RCL to the cartilaginous and ossified capitellum on MRI. The RCL passed through the middle third of the cartilaginous capitellum in almost all cases, whereas its relationship to the ossified capitellum was much less consistent, particularly on the coronal plane [9]. In practical terms, the RCL may be anatomically appropriate relative to the true cartilaginous joint surface while appearing abnormal on a plain radiograph. This problem is most pronounced in young children and on AP radiographs.

**Figure 1:**
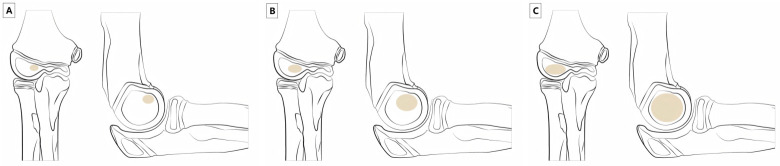
Developmental basis of apparent RCL eccentricity in young children. Panels (**A**–**C**) show schematic early, intermediate, and later ossification stages rather than fixed age thresholds. Capitellar ossification is shown as beginning eccentrically within the cartilaginous capitellum—anteroproximal on the sagittal projection and medial/proximal on the coronal projection—and then enlarging posteriorly, distally, and laterally toward a more central relationship with the cartilaginous capitellum. Consequently, the RCL may appear eccentric relative to the ossified nucleus while still aligning with the cartilaginous joint surface.

Radiographic studies of normal elbows support the same concept. Huang et al. described a consistent eccentric pattern of the RCL in young children, with lateral offset on AP views and posterior offset on lateral views [14]. Baskovic and Gregov further showed that the probability of the RCL crossing the middle third of the ossified capitellum is strongly age- and projection-dependent [11]. Taken together, these studies indicate that the middle-third rule should not be used as an absolute criterion in young children, especially on AP views. Figure 1 summarizes the developmental mechanism underlying apparent RCL eccentricity.

### 4.3. Diagnostic Implications

These developmental findings have direct implications for diagnostic reasoning. First, RCL interpretation is best considered age- and projection-specific. A true lateral radiograph using a radial neck-axis RCL is generally more useful than an AP-based RCL, but even lateral interpretation should be cautious in younger children. Second, RCL deviation relative to the ossified capitellum is not suitable for isolated interpretation. The reader may assess whether the deviation pattern is compatible with developmental eccentricity, whether projection is adequate, and whether there is associated ulnar fracture or plastic deformation.

When ossified landmarks are insufficient or radiographic findings are discordant, the case may be classified as equivocal rather than forced into a normal/abnormal category. This distinction matters clinically: overcalling developmental eccentricity may lead to unnecessary repeat imaging or intervention, whereas undercalling true malalignment may delay reduction. In the imaging interpretation framework proposed in this review, radiographic quality control and age-related ossification assessment therefore precede line selection. If ossified landmarks are inadequate, AP and lateral findings disagree, or clinical suspicion remains high despite a seemingly normal RCL, targeted problem-solving imaging such as ultrasound or MRI may be considered.

Table 2 summarizes pragmatic age-, ossification-, and projection-specific considerations for reporting.

## 5. Radiographic Quality Control Before Drawing Lines

Radiographic line methods are only meaningful when the underlying projection is adequate. In the proposed framework, radiographic quality control is placed before immediate line interpretation. A radiograph that does not include the elbow, a lateral view that is not close to true lateral, or a forearm radiograph that excludes the distal radial physis can make otherwise useful line-based tools unreliable. The proposed framework treats image quality as the first diagnostic gate: if the image cannot support a line-based conclusion, the finding is best reported as limited or equivocal rather than normal.

### 5.1. Minimum Radiographic Requirements

The purpose of imaging in suspected Monteggia lesions is not only to identify an ulnar fracture, but also to evaluate radiocapitellar congruity. Standard elbow AP and lateral radiographs remain the basic views for assessing radiocapitellar alignment in most cases [30]. Forearm radiographs are most informative when they include the elbow whenever Monteggia injury is possible, especially in children with ulnar fracture, ulnar bowing, elbow pain, restricted forearm rotation, or an unclear mechanism of injury. This principle is consistent with the broader pediatric fracture-imaging concept that radiographs should be targeted, child-specific, and sufficient to answer the clinical question rather than narrowly centered only on the most obvious fracture site [34].

This requirement is particularly important in emergency imaging, where radiographs may be centered on the apparent forearm fracture and may not include the elbow adequately. If the elbow is not included, radiocapitellar alignment cannot be confidently assessed. If the study is limited to the forearm shaft, the radiologist should recommend dedicated elbow views rather than infer alignment from incomplete images.

A second field-of-view issue applies to P-line assessment. The P-line is constructed by connecting the midpoints of the proximal and distal radial physes; therefore, a forearm radiograph that excludes the distal radial physis cannot be used for this method [15]. In practical terms, if P-line assessment is intended, the forearm radiograph should include the wrist. When the wrist is not included, the correct next step is repeat or additional imaging rather than approximating the line from incomplete landmarks.

Oblique views may be useful for fracture detection, but they do not replace standard AP and lateral projections for alignment assessment. RCL, P-line, LHL, and ulnar bow sign are projection-dependent geometric tools. A line drawn on an oblique or rotated view may reflect projectional distortion rather than true radiocapitellar malalignment.

### 5.2. Projection, Beam Angle, and Lateral View Quality

The lateral elbow radiograph is central to RCL interpretation, but it must approximate a true lateral view. Pediatric positioning is often difficult because of pain, swelling, immobilization, and limited cooperation. A suboptimal lateral view may change the apparent relationship between the radial neck, radial head, and capitellum.

Iyer et al. emphasized that pediatric elbow trauma imaging usually requires an adequate lateral projection, generally with the elbow flexed and the forearm supinated [30]. This is clinically relevant because the RCL is sensitive to projection and forearm rotation. Kunkel et al. showed that RCL behavior varies with line placement and projection-related factors, including the relationship between radial neck and shaft axes [12]. Thus, when an RCL appears abnormal on a non-true lateral radiograph, the reader should first question the projection before diagnosing radiocapitellar subluxation.

Additional radiographic measurement studies reinforce this point. McCann et al. evaluated how radiographic beam angle affects radiocapitellar ratio measurement, while Sandman et al. studied the effect of elbow and forearm position on radiocapitellar alignment measurements [35,36]. Although these studies addressed radiocapitellar ratio measurements rather than Monteggia diagnosis specifically, they support the broader principle that quantitative assessment of the radiocapitellar relationship is projection-sensitive. Therefore, line-based interpretation is best tied to the quality of the radiograph on which the line is drawn.

A practical reporting principle is that a definitive alignment conclusion should be avoided if the capitellum, radial neck, or radial head is poorly visualized. When these structures are obscured by positioning, cast material, inadequate exposure, or incomplete ossification, the report may explicitly state that radiocapitellar assessment is limited and suggest repeat standardized views or targeted problem-solving imaging.

### 5.3. AP Projection and Coronal-Plane Assessment

AP radiographs are important for detecting lateral displacement and coronal-plane malalignment, but AP-based RCL interpretation is particularly vulnerable in children. Eccentric ossification of the capitellum may make the ossified nucleus appear offset relative to the cartilaginous capitellar center [9,10]. AP projectional rotation can further distort this relationship.

For this reason, AP RCL is not suitable as a standalone criterion for confirming or excluding a Monteggia lesion in children. When coronal-plane malalignment is suspected, particularly in possible Bado type III or lateral displacement patterns, LHL may provide a more appropriate adjunctive assessment, provided the AP radiograph is adequate [16]. If the AP view is rotated or the lateral ossification margin of the distal humerus is poorly visible, LHL interpretation should also be reported as limited.

### 5.4. When Radiographs Should Be Considered Equivocal

In the proposed framework, a radiograph may be classified as inadequate or equivocal for line-based diagnosis when the elbow is not included, when AP and lateral views are unavailable, when the lateral view is not close to true lateral, when the capitellum or radial neck is poorly visualized, when the forearm radiograph excludes the distal radial physis despite planned P-line assessment, or when imaging findings are discordant with clinical suspicion.

In these situations, the preferred next step is repeat standardized radiography when feasible. If repeat radiography remains limited because of age, pain, incomplete ossification, or clinical urgency, the case may proceed to targeted problem-solving imaging. Ultrasound can assess cartilaginous radiocapitellar congruity dynamically and without radiation. MRI can clarify cartilage alignment and soft tissue interposition. Arthrography may be reserved for selected young children requiring intraoperative confirmation.

This quality-control step shifts the diagnostic process away from “drawing a line at all costs” toward asking whether the image can support the conclusion being made. In children, especially younger patients, an equivocal radiograph should be treated as a diagnostic state requiring further assessment, not as a negative study. The practical quality checklist is summarized in Box 1.

Box 1 **Radiographic quality checklist before line interpretation.**

- Elbow included when Monteggia lesion is suspected?
- AP and lateral elbow projections available, or limitation explicitly stated?
- Lateral view close to true lateral?
- Capitellum, radial neck, and radial head sufficiently visible for the intended line/sign assessment?
- Distal radial physis included if P-line assessment is planned?
- Imaging findings concordant with the clinical scenario?

If one or more criteria are not met, repeat standardized views or use targeted problem-solving imaging rather than making a definitive line-based diagnosis.

## 6. Diagnostic Tools by Imaging Scenario

After radiographic quality has been verified, diagnostic tools may be selected according to the available imaging scenario rather than applied indiscriminately. In suspected pediatric Monteggia lesions, the appropriate method depends on the child’s age, projection quality, whether dedicated elbow views are available, whether the wrist is included on forearm radiographs, and whether ulnar plastic deformation is suspected. A scenario-based approach may help avoid both overinterpretation of developmental eccentricity and under-recognition of true radiocapitellar malalignment, although this has not been validated in prospective reader studies.

### 6.1. Standard Lateral Elbow Radiograph: Radial Neck-Axis RCL

The radiocapitellar line (RCL) remains the most widely used screening tool for radiocapitellar alignment. It is traditionally drawn along the long axis of the radius and extended proximally to determine whether it intersects the capitellum. However, the axis chosen for drawing the line matters. Studies of normal pediatric elbows suggest that a line based on the radial neck axis on a true lateral view is more consistent than a shaft-based line [12,13].

Kunkel et al. showed that the RCL may fail to pass through the middle third of the ossified capitellum even in normal children, with variability related to projection, line placement, and visibility of the capitellum [12]. Ramirez et al. further reported that a shaft-based line can miss the capitellum in a notable proportion of normal pediatric elbow radiographs, supporting the radial neck rather than the radial shaft as the preferred reference axis [13]. On adequate lateral elbow radiographs, a radial neck-axis RCL may be the most appropriate RCL construction method, based on normal-elbow alignment studies.

Nevertheless, the RCL is best interpreted as a screening sign rather than a definitive diagnostic test. Its relationship to the ossified capitellum is strongly age-dependent [11]. MRI evidence further shows that the RCL may align with the cartilaginous capitellum even when it appears eccentric relative to the ossified capitellar nucleus [9,10]. Therefore, an apparently abnormal RCL in a young child or on a suboptimal projection may prompt reassessment of image quality and integration with ulnar morphology, rather than immediate diagnosis of dislocation.

### 6.2. Forearm Radiograph-Only Scenario: P-Line

A common clinical scenario is that the child is imaged primarily for forearm trauma, with radiographs centered on the forearm rather than on the elbow. In such cases, standard elbow views may be unavailable, and applying a conventional RCL to a forearm radiograph may be unreliable. The P-line was proposed to address this scenario.

The P-line is constructed by connecting the midpoints of the proximal and distal radial physes and extending the line proximally toward the capitellum [15]. Wang and Su evaluated this method on pediatric forearm radiographs and found that it more consistently intersected the capitellum than traditional RCL methods based on the radial neck or shaft, particularly on AP forearm views [15]. Therefore, when only forearm radiographs are available, the P-line may serve as a useful preliminary tool for radiocapitellar alignment assessment.

This method has a key technical prerequisite: both proximal and distal radial physes must be visible. A forearm radiograph excluding the wrist cannot be used for P-line assessment. The P-line is therefore not a replacement for dedicated elbow radiographs; rather, it may be used as a scenario-specific screening method when complete forearm radiographs are available. If the P-line is abnormal, if ulnar bowing is present, or if clinical suspicion remains high, dedicated elbow AP and lateral radiographs may be required.

### 6.3. AP or Coronal-Plane Concern: LHL and Other Adjunctive Lines

AP radiographs are important for detecting coronal-plane displacement, particularly in suspected lateral displacement patterns such as Bado type III lesions. However, AP-based RCL interpretation is limited in children because the RCL may appear eccentric relative to the ossified capitellum even in normal elbows [9,10,16]. In this setting, the lateral humeral line (LHL) provides an additional coronal-plane reference.

The LHL is drawn along the lateral edge of the ossified distal humerus on the AP radiograph, parallel to the humeral shaft. Its relationship to the lateral cortex of the radial neck is then assessed [16]. In normal elbows, the LHL should lie along or lateral to the lateral border of the radial neck. When the radial head is laterally displaced, particularly in Bado type III patterns, the LHL may pass through or medial to the radial neck [16].

Other AP-based adjuncts have also been explored. Tan et al. proposed the radiocoronoid line as an additional method for assessing lateral elbow dislocation in the AP projection and reported higher reliability than the RCL in that setting [37]. This line may be considered an additional problem-solving tool when AP/coronal-plane malalignment is suspected. However, the current review keeps LHL as the principal AP adjunct in the proposed Monteggia framework because LHL has been specifically discussed in relation to pediatric radiocapitellar alignment and Bado type III patterns. The radiocoronoid line is best treated as a supplementary option until further Monteggia-specific validation becomes available.

### 6.4. Ulnar Plastic Deformation: Ulnar Bow Sign

Monteggia lesions are best considered ulna–radial head injury units rather than isolated radiocapitellar alignment abnormalities. This is particularly important in children, in whom the ulna may be plastically deformed without a clear fracture line. For this reason, ulnar morphology may be assessed together with any radiocapitellar line method.

Lincoln and Mubarak described the ulnar bow sign in cases initially interpreted as isolated traumatic radial-head dislocation but associated with ulnar plastic deformation [17]. The measurement is performed on a true lateral forearm radiograph by drawing a reference line along the dorsal border of the ulna from the olecranon to the distal ulnar metaphysis and measuring the maximum perpendicular distance from the ulnar border to this line. A maximum ulnar bow greater than 1 mm has been used as a practical threshold suggesting plastic deformation [3,17].

The value of the ulnar bow sign lies in its ability to identify the ulnar component of the injury. If radiocapitellar alignment is abnormal and ulnar bowing is present, suspicion for a Monteggia lesion or Monteggia-equivalent injury is high. If alignment appears abnormal but ulnar morphology is normal, the reader may first recheck projection quality, line placement, and developmental eccentricity before diagnosing true dislocation. Conversely, if alignment appears acceptable but ulnar bowing is obvious or clinical suspicion is strong, occult radiocapitellar subluxation should remain in the differential diagnosis.

Monteggia-equivalent lesions further support this combined approach. Classic and contemporary reviews of Monteggia-equivalent injuries emphasize that pediatric variants may include subtle ulnar deformation, atypical forearm injury patterns, or associated radial head/neck injuries rather than the complete fracture-dislocation pattern described in classic Bado types [7,8]. These variants reinforce the need to interpret ulnar morphology and radiocapitellar alignment together rather than relying on a single line.

Quantitative ulnar bow assessment may also support surgical communication in missed or chronic cases. Liao et al. reported that ulnar bow parameters may correlate with surgical planning in missed Bado type I injuries [18]. Although this is more relevant to delayed presentations than acute screening, it reinforces the importance of evaluating the ulna as part of the diagnostic process.

### 6.5. Equivocal Radiographs: Ultrasound, MRI, and Arthrography

Radiographs may be classified as equivocal when ossified landmarks are insufficient, projections are suboptimal, line methods are discordant, or clinical suspicion remains high despite apparently normal alignment. In such cases, the goal should not be to force a binary radiographic interpretation, but to select the problem-solving modality that best answers the unresolved question.

Ultrasound is particularly useful in younger children because it can visualize cartilaginous structures dynamically and without radiation. Cepelik et al. described sonographic signs of radiocapitellar congruity, including the double-hump sign and the congruency sign [19]. The double-hump sign reflects the adjacent rounded contours of the radial head cartilage and capitellar cartilage, whereas the congruency sign reflects the matched concave–convex relationship of the radial articular surface and capitellum [19]. Ultrasound may be useful as a targeted problem-solving modality in centers with appropriate pediatric musculoskeletal expertise, particularly for low ossification cases, discordant radiographic findings, or post-reduction assessment.

MRI provides direct visualization of the cartilaginous capitellum and radial head and may help resolve cases in which the RCL appears abnormal relative to the ossified nucleus but developmental anatomy suggests uncertainty [9,10]. MRI may also help assess soft tissue causes of persistent malalignment or instability. Tan et al. described annular ligament pathology in pediatric Monteggia fractures, including interposition that may affect reduction and stability [21]. Thus, MRI is most useful when cartilage alignment or soft tissue interposition requires clarification.

Arthrography has a narrower but important role. Lee et al. showed that elbow arthrography can clarify occult radial head subluxation or dislocation in young children with ulnar fracture when ossification is insufficient for reliable radiographic interpretation [20]. It may be reserved for selected young children with insufficient ossification, particularly when intraoperative confirmation of radiocapitellar alignment or reduction quality is needed.

Taken together, targeted problem-solving imaging may be selected according to the unresolved diagnostic question and the practical tradeoffs of each modality. Repeat standardized radiographs are most appropriate when projection is inadequate; contralateral comparison should be limited to selected low-ossification cases because it adds radiation and normal bilateral anatomy is not perfectly symmetric; ultrasound is useful only where pediatric musculoskeletal expertise is available; MRI is best reserved for cartilage, marrow, ligament, or soft-tissue questions; arthrography is invasive and usually procedural; and CT is primarily a bony problem-solving tool rather than a solution for cartilaginous incongruity.

### 6.6. Differential Considerations and the Role of CT

Several conditions can mimic or obscure pediatric Monteggia lesions. Congenital radial head dislocation is usually associated with chronic or bilateral findings, radial head dysplasia, and absence of an acute ulnar injury. Isolated radial neck fracture may alter the proximal radial appearance without the ulna–radial head injury unit typical of Monteggia lesions. True elbow dislocation is uncommon in younger children and may be distinguished from radiocapitellar malalignment associated with ulnar fracture or bowing. Nursemaid’s elbow is primarily a clinical diagnosis in younger children and usually lacks traumatic ulnar deformity or radiographic fracture. These distinctions reinforce the value of interpreting radiocapitellar alignment together with ulnar morphology, injury mechanism, and age-related ossification [7,8,30].

CT is not emphasized as a routine first problem-solving tool in this framework because it involves ionizing radiation and does not directly solve the main diagnostic problem in low ossification children: non-visualization of cartilaginous articular structures. CT may be useful in selected complex fractures or for preoperative assessment of bony anatomy, but ultrasound and MRI are generally more appropriate when the unresolved question is cartilaginous radiocapitellar congruity or soft-tissue interposition [30].

Table 3 summarizes problem-solving imaging options for unresolved alignment questions.

Table 4 summarizes scenario-based line and sign methods.

### 6.7. Practical Hierarchy for Discordant Alignment Findings

Discordance among RCL, P-line, LHL, ulnar bow sign, and clinical suspicion should not be resolved by a simple majority vote among line methods. Each method answers a different imaging question and depends on different anatomic landmarks. When findings are discordant, interpretation should first return to image adequacy and ossification stage. If the elbow is incompletely included, the lateral view is not close to true lateral, or the required landmarks are not visible, the study should be reported as limited or equivocal rather than normal.

**Figure 2:**
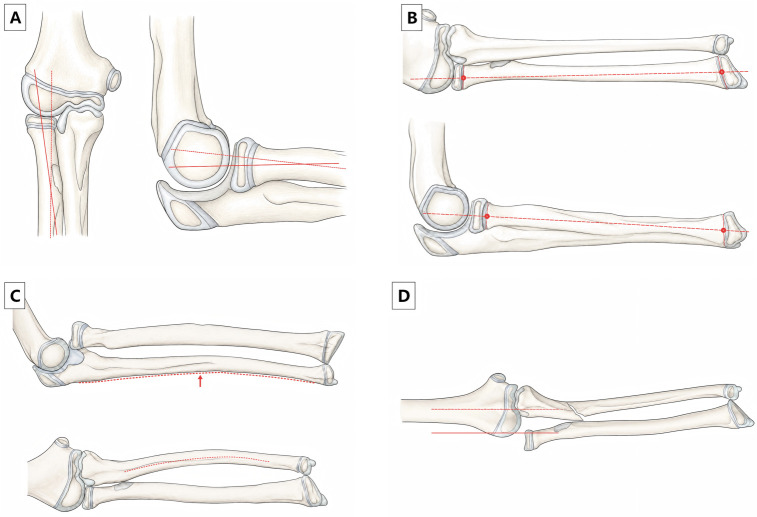
Scenario-specific line and sign methods for suspected pediatric Monteggia lesions. (**A**) RCL on AP and lateral radiographs, showing shaft-axis and radial neck-axis placement; on adequate lateral elbow views, the neck-axis construction is generally preferred. (**B**) P-line connects the midpoints of the proximal and distal radial physes and requires a complete forearm radiograph including the wrist. (**C**) Ulnar bow sign/maximum ulnar bow (MUB) is assessed on true lateral forearm radiographs; the commonly cited 1 mm threshold is a warning sign, not a universal cutoff. (**D**) LHL is drawn along the lateral margin of the distal humerus on AP radiographs and is limited when rotation or incomplete ossification obscures the lateral margin. Red solid and dashed lines indicate alternative reference axes or measurement baselines; red circles mark physeal midpoint landmarks; and the red arrow indicates the site of maximum ulnar bow measurement.

Figure 2 summarizes the line and sign drawing methods used in the scenario-specific assessment.

When adequate elbow radiographs are available, a true lateral view with a radial neck-axis RCL is generally the preferred first-line alignment assessment. AP-based RCL interpretation is more vulnerable to eccentric capitellar ossification and projection effects, particularly in young children. P-line assessment is most useful in the forearm-radiograph scenario, but only when both proximal and distal radial physes are visible. LHL is an AP/coronal-plane adjunct, particularly when lateral displacement is suspected, but should not be treated as an independent diagnostic arbiter when lateral distal humeral ossification or the radial neck cortex is poorly visualized. Ulnar bowing or proximal ulnar injury should increase concern even when an isolated line appears reassuring. Persistent clinical concern, ulnar bowing, or unresolved discordance should prompt repeat standardized radiographs or targeted problem-solving imaging rather than a definitive normal report.

Table 5 summarizes the practical hierarchy for discordant alignment findings.

## 7. Diagnostic Errors and Reader Performance

Missed pediatric Monteggia lesions usually reflect failure to integrate several subtle findings rather than absence of a single radiographic sign. Ulnar plastic deformation, incomplete ossification, non-dedicated forearm radiographs, projection-related distortion, and mild radiocapitellar malalignment may each appear minor when interpreted in isolation. Together, however, they form a diagnostic pattern that may prompt repeat standardized imaging, targeted problem-solving imaging, or orthopedic review.

Monteggia-specific observer-performance data support this concern. Choi et al. evaluated interpretation of pediatric elbow and forearm injuries among physicians with different specialties and experience levels; missed diagnosis was associated with recognition failure, subtle radiographic findings, ulnar plastic deformation, and lack of dedicated elbow imaging [6]. Broader musculoskeletal radiography literature also supports deliberate search strategies and awareness of perceptual or cognitive bias when subtle fractures or alignment abnormalities are present [38,39].

For this reason, the proposed framework is best viewed as a structure for documenting image adequacy, developmental limitations, line/sign selection, ulnar morphology, and the unresolved diagnostic question. It is not a validated error-reduction intervention. Its practical role is to make explicit the minimum interpretive questions to address before a suspected Monteggia lesion is reported as showing no imaging evidence of radiocapitellar malalignment on adequate views.

## 8. Proposed Imaging Interpretation Framework

**Figure 3:**
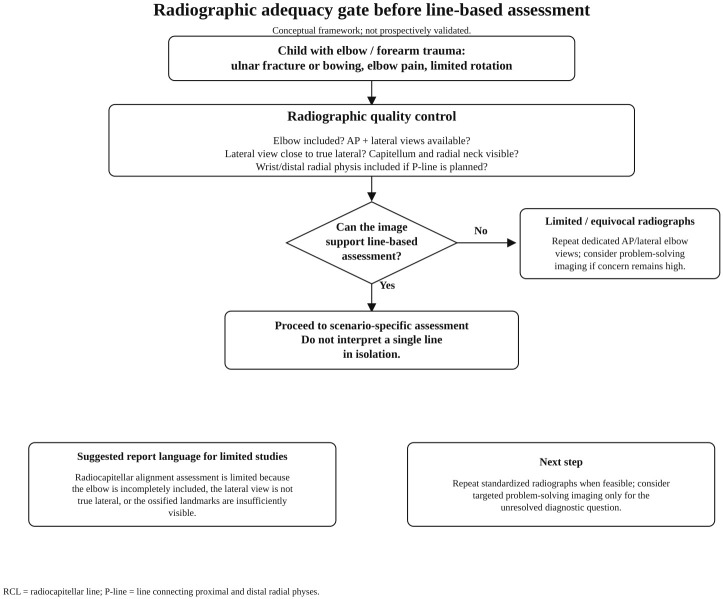
Radiographic adequacy gate before line-based assessment. This is a conceptual framework component and has not been prospectively validated. The first step is to determine whether the radiographs can support line-based interpretation. If the elbow is not included, projection is inadequate, ossified landmarks are not visible, or the distal radial physis is missing when P-line assessment is planned, the study is best reported as limited or equivocal rather than normal.

**Figure 4:**
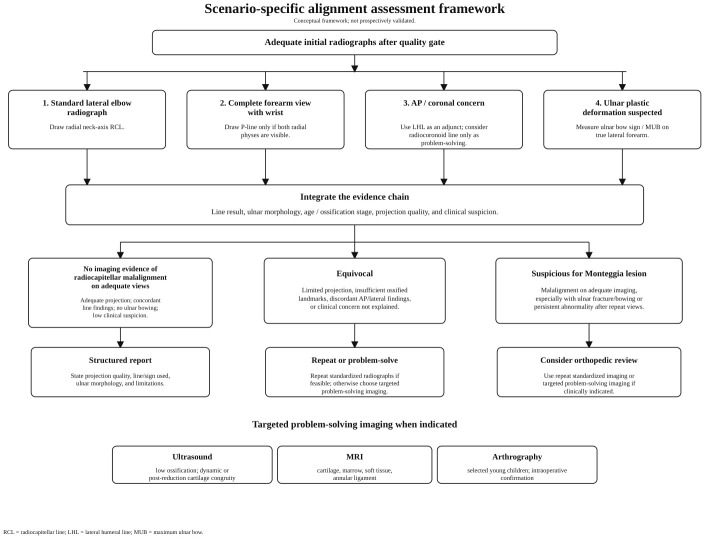
Scenario-specific alignment assessment framework for suspected pediatric Monteggia lesions. This conceptual framework is intended for image interpretation and reporting support and has not been prospectively validated. After the radiographic adequacy gate is satisfied, assessment is selected according to scenario and integrated with ulnar morphology, age/ossification stage, projection quality, and clinical suspicion. The categories shown are qualitative framework outputs, not validated diagnostic classes.

The proposed framework integrates the evidence reviewed above into a practical sequence for radiographic interpretation and reporting. It is intended to make the evidence chain explicit rather than to function as a validated clinical prediction rule. The first step is a radiographic adequacy gate (Figure 3); only studies that can support line-based interpretation may proceed to scenario-specific assessment (Figure 4).

### Operational Definitions for the Proposed Framework

The proposed framework is qualitative and should not be interpreted as a numeric diagnostic rule or a validated clinical prediction rule. “Abnormal alignment” refers to an RCL or adjunctive line that remains inconsistent with expected age- and projection-specific alignment after radiographic quality has been verified. “Ulnar bowing” refers to measurable dorsal ulnar bowing on a true lateral forearm radiograph; the commonly cited MUB > 1 mm threshold should be interpreted as a practical warning sign rather than a universal diagnostic cutoff. “High clinical suspicion” refers to an injury mechanism or examination inconsistent with an isolated forearm fracture, including elbow pain, restricted forearm rotation, proximal ulnar injury, visible ulnar plastic deformation, or discordance between symptoms and apparent alignment.

**Pitfall checklist.** Do not exclude a Monteggia lesion when the elbow is incompletely included, the lateral view is not close to true lateral, ossified landmarks are insufficiently visible, the ulna shows plastic deformation, AP and lateral findings are discordant, or clinical suspicion persists despite apparently maintained alignment. In these settings, report the limitation and consider repeat standardized radiographs or targeted problem-solving imaging according to the unresolved question.

Table 6 summarizes the operational categories used in the proposed framework.

Table 7 provides an everyday-use summary for common clinical imaging scenarios.

## 9. Structured Reporting Template

A structured report can translate the framework into a reproducible communication tool. The report should not merely state that radiocapitellar alignment is maintained; it should describe the adequacy of the projection, the line or sign used, ulnar morphology, limitations, and the recommended next step when the study is indeterminate.

Table 8 summarizes suggested reporting elements for suspected pediatric Monteggia lesions.

## 10. Future Directions: Explainable Automated Assessment

AI-assisted interpretation of pediatric elbow trauma is an emerging area, but its relevance to Monteggia lesions should be interpreted cautiously. Generic pediatric elbow fracture classifiers and multimodal language models may detect fractures or broad abnormalities, but Monteggia recognition requires a different task: determining whether the available radiographs are adequate, identifying the correct age- and projection-dependent landmarks, drawing alignment lines appropriately, assessing ulnar morphology, and expressing uncertainty when findings are discordant.

General pediatric elbow AI studies provide useful background, but they do not directly validate Monteggia alignment assessment [26,27,28]. The most relevant current direction is Monteggia-specific automation: Chakladar et al. developed a pipeline combining segmentation-based assessment of radiocapitellar alignment with detection of associated ulnar fracture [29]. A clinically useful tool would ideally perform several linked tasks rather than a single fracture/no-fracture classification. These include detection of elbow inclusion and projection adequacy, automated placement of radial neck-axis RCL and P-line when landmarks are visible, assessment of LHL or other AP/coronal adjuncts when lateral displacement is suspected, measurement of ulnar bowing on true lateral forearm views, and generation of an “equivocal” output when image quality or ossification does not support a confident interpretation.

At present, AI should be regarded as a potential measurement and triage aid rather than a replacement for expert interpretation. Prospective validation should evaluate not only classification accuracy but also whether AI-supported measurement reduces missed Monteggia lesions without increasing false-positive diagnosis in young children with physiologic ossification-related RCL eccentricity. Explainable visual overlays will be essential so that readers can evaluate whether automated landmarks are anatomically plausible.

## 11. Discussion

This narrative review synthesizes evidence from pediatric elbow ossification studies, radiographic line-method studies, missed Monteggia reports, targeted problem-solving imaging studies, observer-performance research, and emerging automated image interpretation. The central message is that radiocapitellar alignment in children should not be interpreted using a single binary line rule. Instead, interpretation may begin with radiographic adequacy, then incorporate developmental anatomy, scenario-specific line or sign selection, ulnar morphology, and the unresolved clinical question.

The RCL remains useful as a first-line screening sign, but normal-elbow studies and MRI data show why its relationship to the ossified capitellum can be misleading in young children, particularly on AP views [9,10,11,12,13,14]. A radial neck-axis RCL is most appropriate on a true lateral elbow radiograph; P-line is a preliminary tool for complete forearm radiographs; and LHL or other AP/coronal-plane adjuncts can be considered when lateral displacement is suspected [15,16,37]. These tools are best interpreted as components of an evidence chain rather than stand-alone diagnostic rules.

The inference from evidence to framework is therefore deliberately limited. Normal-elbow and developmental studies support the claim that single-line interpretation can be misleading; Monteggia-related missed-injury literature supports attention to the ulna–radial head injury unit; problem-solving imaging studies support targeted escalation when radiographs remain unresolved; and observer-performance or automation studies support structured search and reporting. None of these evidence streams proves that the proposed framework improves diagnostic accuracy. The framework is consequently presented as an interpretive and reporting structure that requires prospective reader-performance validation.

Ulnar morphology is central because Monteggia lesions are ulna–radial head injury units, not isolated radiocapitellar alignment abnormalities. Ulnar bow sign and Monteggia-equivalent literature support consideration of radiocapitellar alignment with assessment for ulnar fracture, bowing, or plastic deformation [7,8,17,18]. When radiographs are equivocal, targeted problem-solving imaging may be selected according to the unresolved question: ultrasound for dynamic cartilaginous congruity, MRI for cartilage or soft-tissue interposition, and arthrography for selected young children requiring intraoperative or anesthesia-based confirmation [19,20,21].

Although this review focuses on diagnosis rather than treatment, delayed recognition may lead to chronic radial head dislocation, deformity, limited motion, pain, nerve symptoms, and more complex reconstruction [2,3,4,5,40,41]. The proposed framework is intended to support clearer reporting of suspicious or insufficiently characterized cases, not to replace orthopedic judgment or local imaging protocols. Future observer-performance studies should test whether framework-informed interpretation improves sensitivity, specificity, interobserver agreement, confidence, repeat-imaging recommendations, and clinical management.

## 12. Take-Home Messages for Clinical Practice

For emergency physicians, the main practical message is that a pediatric forearm fracture should not be interpreted without confirming whether the elbow has been adequately included and whether radiocapitellar alignment can be assessed. Ulnar bowing, elbow pain, or restricted forearm rotation should lower the threshold for dedicated elbow radiographs or orthopedic review.

For radiologists, the key principle is to assess image adequacy before drawing lines. In young children, RCL behavior is influenced by ossification stage and projection; the middle-third rule should not be applied rigidly to the ossified capitellum. Line or sign selection should be scenario-specific, and discordant findings should be reported as limited or equivocal rather than forced into a binary normal/abnormal conclusion.

For pediatric orthopedic surgeons, the framework may help identify when a seemingly reduced or minimally displaced injury still requires further assessment. Persistent clinical concern, ulnar plastic deformation, or unresolved radiographic discordance may justify repeat standardized radiographs, dynamic assessment, ultrasound, MRI, or arthrography depending on the unresolved diagnostic question.

## 13. Applicability and Limitations

The practical contribution of this review is its scenario-based organization of evidence that is often discussed separately: pediatric elbow ossification, RCL limitations, P-line and LHL assessment, ulnar bow sign, ultrasound, MRI, arthrography, diagnostic performance, and automated interpretation. This organization reflects how pediatric elbow and forearm radiographs are encountered in practice and provides a framework for reporting, teaching, multidisciplinary communication, and future validation studies.

Several limitations are relevant. This is a narrative review rather than a protocol-driven systematic or scoping review. Although the literature was selected to cover the major diagnostic domains relevant to pediatric Monteggia lesions, formal database-level PRISMA screening, risk-of-bias assessment, and pooled diagnostic accuracy estimates were not performed. The available studies are heterogeneous in design, population, age range, imaging modality, projection requirements, and outcome measures. Many line-method studies were performed in normal elbows; therefore, their findings support limitations and potential pitfalls of line interpretation but do not directly provide sensitivity or specificity for Monteggia lesions.

Second-line imaging evidence is also limited. Ultrasound studies are affected by operator dependence and relatively small cohorts [19]. Arthrography studies are limited by sample size and institutional practice patterns, and the technique is invasive [20]. MRI provides valuable information on cartilage and soft tissue, but it may not be practical in all acute settings because of availability, cost, and sedation considerations in younger children [9,10,21]. Finally, although emerging AI and automated measurement tools are promising, they require broader external validation across age groups, institutions, acquisition protocols, and injury patterns before routine deployment [26,27,28,29]. This review did not include a prospective patient cohort or newly collected institutional imaging dataset; therefore, the figures are intended as interpretive and educational examples rather than validation of the proposed framework. Additional historical, missed-injury, chronic-lesion, and general pediatric elbow imaging sources retained in the 57-record literature set are summarized in Supplementary Table S1 to maintain traceability of the narrative evidence base.

## 14. Conclusions

Radiocapitellar alignment assessment in suspected pediatric Monteggia lesions requires more than drawing a single line. In young children, incomplete ossification and eccentric capitellar development can make the relationship between the RCL and the ossified capitellum misleading. We propose an imaging interpretation framework that begins with radiographic quality control and then applies line methods according to the imaging scenario: radial neck-axis RCL on true lateral elbow radiographs, P-line on complete forearm radiographs, and LHL or other AP/coronal-plane adjuncts when lateral displacement is suspected. Ulnar bow sign may be considered within an integrated assessment because subtle ulnar plastic deformation may be the key clue to a Monteggia-equivalent injury.

When radiographs are equivocal, repeat standardized radiographs or targeted problem-solving imaging may be selected according to the unresolved diagnostic question. Ultrasound, MRI, and arthrography have distinct problem-solving roles rather than being interchangeable tests. The framework proposed here may help structure interpretation and reporting, but it should not replace local imaging protocols, orthopedic judgment, or formal validation. Prospective observer-performance studies are required to determine whether it improves diagnostic accuracy, reporting consistency, or clinical management.

## Figures and Tables

**Table 1:** Evidence-to-framework linkage for radiocapitellar alignment assessment. The table emphasizes evidence directness and representative sample context; it does not imply that all tools have Monteggia-specific diagnostic accuracy data.

Framework Component	Representative Evidence and Basic Data	Directness Category	Main Contribution	Major Limitation for This Framework
RCL and ossification-related pitfalls	Fader et al. [9,10]; Kunkel et al. [12]; Ramirez et al. [13]; Huang et al. [14]; Baskovic and Gregov [11]	Indirect normal-elbow/ developmental evidence	Demonstrates age-, ossification-, projection-, and line-placement dependence of RCL; supports caution with a middle-third rule in young children.	Does not provide Monteggia-specific sensitivity, specificity, or outcome benefit of a framework.
P-line for forearm radiographs	Wang and Su [15]; 170 normal pediatric forearm radiographic examinations reported in the method study	Projection or measurement- method evidence	Provides a forearm-radiograph method when both proximal and distal radial physes are visible.	Validated mainly in normal forearm radiographs; not a substitute for dedicated elbow views.
LHL and AP/coronal-plane adjuncts	Souder et al. [16]; 37 normal AP elbow radiographs with Bado III comparison cases as reported	Projection or measurement- method evidence with limited Monteggia comparison	Provides an AP/coronal-plane adjunct when AP RCL is unreliable or lateral displacement is suspected.	Small method study; AP rotation and incomplete lateral ossification margins may limit interpretation.
Ulnar bow sign/MUB	Lincoln and Mubarak [17]; Kemnitz et al. [25]; Liao et al. [18]	Monteggia-related clinical/missed-injury evidence	Emphasizes ulnar plastic deformation as part of the injury unit and a clue to occult Monteggia-equivalent injury.	Requires true lateral whole-forearm imaging; MUB threshold is a warning sign rather than a universal diagnostic cutoff.
Ultrasound	Cepelik et al. [19]; prospective problem-solving diagnostic study of pediatric elbow ultrasonography	Problem-solving imaging evidence	Visualizes cartilaginous congruity and may help in low-ossification or post-reduction assessment.	Operator- and position-dependent; does not replace radiographs for fracture characterization.
MRI	Fader et al. [9,10]; Tan et al. [21]	Problem-solving imaging evidence	Clarifies cartilage, ossification–center relationship, marrow, annular ligament, and soft-tissue interposition.	Availability, cost, scan time, and sedation considerations limit routine acute use.
Arthrography	Lee et al. [20]; small series of children with ulnar fracture and occult radial head subluxation	Problem-solving imaging evidence	Clarifies occult subluxation in selected young children with insufficient ossification, often in procedural settings.	Invasive and institutional-practice dependent; not a routine screening test.
Diagnostic error and automation	Choi et al. [6]; observer-performance study; generic pediatric elbow AI studies [26,27,28]; Chakladar et al. [29] Monteggia-specific automation	Observer-performance/ contextual evidence and AI/ automation development evidence	Supports structured search, cautious reporting, and future explainable measurement tools.	Does not prospectively validate the proposed framework or replace human clinical judgment.

**Table 2:** Age-, ossification-, and projection-specific interpretation considerations for radiocapitellar alignment. These categories are pragmatic interpretive considerations rather than validated diagnostic thresholds.

Age/Ossification Context	Main Interpretive Risk	More Informative Assessment	Acceptable Uncertainty	When Not to Conclude Normal Alignment
Young child or low ossification	Ossified capitellum may not represent the cartilaginous center; AP RCL may appear eccentric.	Radiographic adequacy check; lateral radial neck-axis RCL when possible; ulnar morphology; consider US/MRI if unresolved.	RCL eccentricity relative to the ossified nucleus may be developmental, especially on AP/coronal assessment.	Non-true lateral view, poor landmark visibility, ulnar bowing/fracture, AP/lateral discordance, or persistent clinical concern.
Partial ossification/ school-age child	Landmarks are more visible but projection, rotation, and line-axis selection remain important.	True lateral radial neck-axis RCL with AP/coronal adjuncts (LHL or radiocoronoid line) when lateral displacement is suspected.	Mild line discordance should be interpreted with projection quality and ulnar morphology rather than in isolation.	Forearm-only imaging without elbow, wrist absent when P-line is needed, non-true lateral view, or discordant line/sign findings.
Older child/adolescent or more mature ossification	Line methods become more adult-like, but ulnar plastic deformation or subtle malalignment may still be missed.	Standard elbow AP/lateral radiographs; radial neck-axis RCL; LHL/P-line/MUB according to imaging scenario.	Less uncertainty is expected when projections and landmarks are adequate, but clinical discordance still matters.	Ulnar bowing, persistent malalignment after repeat standardized views, or symptoms inconsistent with an isolated forearm fracture.

**Table 3:** Problem-solving imaging options when radiocapitellar alignment remains unresolved.

Unresolved Question	Possible Modality	Evidence Basis	Key Limitation	Not Intended for
Incomplete or non-standard radiographs	Repeat standardized radiographs	Practice guidance and radiographic-quality principles	Pain, immobilization, and cooperation may still limit positioning	Replacing problem-solving imaging when cartilage or soft tissue remains the unresolved question
Uncertain ossification or symmetry	Contralateral comparison radiographs	Practice-resource suggestion and pediatric elbow developmental context	Additional radiation and imperfect symmetry; use should be targeted	Routine use when ipsilateral views are already adequate and concordant
Reduction or stability concern during orthopedic management	Fluoroscopy/dynamic assessment	Practice-resource and intraoperative problem-solving context	Operator-dependent; often in orthopedic or procedural setting	Routine screening of uncomplicated radiographs
Low ossification or dynamic cartilage congruity question	Ultrasound	Problem-solving imaging evidence in children	Operator- and position-dependent; requires appropriate expertise	Replacing standard radiographs for fracture characterization
Cartilage, marrow, annular ligament, or soft-tissue interposition	MRI	Developmental MRI and soft-tissue pathology evidence	Availability, cost, scan time, and possible sedation	Routine first-line acute screening
Occult subluxation in selected young children, especially during anesthesia or surgery	Arthrography	Small problem-solving series in ulnar fracture with uncertain alignment	Invasive; institutional practice dependent	Routine noninvasive screening
Complex osseous injury or preoperative bony anatomy	CT	General pediatric trauma imaging context	Ionizing radiation; does not directly solve cartilaginous non-ossification	Low-ossification cartilage congruity questions when US/MRI is more appropriate

**Table 4:** Scenario-based assessment tools for suspected pediatric Monteggia lesions. Evidence directness and major limitations are summarized here and in Supplementary Table S1.

Imaging Scenario	Primary or Adjunctive Assessment	Evidence Type	Key Limitations	Suggested Next Step If Equivocal
Standard lateral elbow radiograph	Radial neck-axis RCL	Normal pediatric elbow alignment studies; indirect Monteggia relevance	Age-, projection-, and technique-dependent; shaft-axis lines may be misleading	Recheck projection quality; assess ulnar bow; consider US/MRI if discordant
Forearm radiograph with wrist included	P-line	Normal pediatric forearm radiograph method study	Requires visible proximal and distal radial physes; not a substitute for dedicated elbow views	Obtain dedicated elbow AP/lateral views if abnormal or incomplete
AP/coronal-plane concern	LHL; radiocoronoid line as optional adjunct	Small method studies; limited Monteggia-specific validation	AP rotation and unclear ossification margins may limit interpretation	Correlate with lateral view and ulnar morphology
Ulnar plastic deformation suspected	Ulnar bow sign/MUB	Classic diagnostic sign and missed-injury literature	Requires true lateral whole-forearm view; MUB threshold is a warning sign, not a universal cutoff	Combine with alignment findings; escalate if clinical concern persists
Low ossification or discordant radiographs	Ultrasound	Prospective diagnostic/problem-solving study	Operator- and position-dependent	MRI if still indeterminate or soft-tissue question persists
Persistent uncertainty or soft-tissue concern	MRI	Developmental MRI evidence and soft-tissue pathology studies	Availability, cost, and possible sedation in young children	Use for cartilage/soft-tissue clarification and orthopedic decision-making
Young child requiring intraoperative confirmation	Arthrography	Small arthrography problem-solving series	Invasive; not routine screening	Confirm reduction, congruity, and stability intraoperatively

**Table 5:** Practical hierarchy for discordant radiocapitellar alignment findings. This hierarchy is intended for interpretation and reporting; it is not a validated diagnostic scoring system.

Step	Question	Preferred Action	Reporting Implication
1	Are the images adequate?	Check elbow inclusion, AP/lateral views, true lateral quality, visible capitellum/radial neck, and wrist/distal radial physis if P-line is planned.	If inadequate, report the assessment as limited or equivocal rather than normal.
2	Is a standard lateral elbow view available?	Use radial neck-axis RCL as the primary line-based assessment when landmarks are visible.	Avoid rigid application of the ossified-capitellum middle-third rule in young children.
3	Is the study a complete forearm radiograph?	Use P-line only if proximal and distal radial physes are both visible.	Abnormal or unavailable P-line should prompt dedicated elbow views when clinical concern persists.
4	Is AP/coronal lateral displacement suspected?	Use LHL as an adjunct; consider radiocoronoid line only as problem-solving.	LHL is limited if the lateral distal humeral margin or radial neck cortex is poorly visualized.
5	Is ulnar plastic deformation present?	Describe ulnar bowing or MUB on a true lateral forearm radiograph.	Ulnar bowing increases suspicion even when one alignment line appears reassuring.
6	Are findings discordant or clinically inconsistent?	Repeat standardized views or use US/MRI/arthrography according to the unresolved question.	Do not force a normal/abnormal binary conclusion.

**Table 6:** Operational categories used in the proposed imaging interpretation framework. These qualitative categories are intended to support consistent reporting and triage language, not to function as a validated prediction rule.

Category	Operational Meaning	Suggested Reporting or Next Step
Radiographically adequate	Elbow included; AP and lateral views available; lateral view close to true lateral; capitellum/radial neck sufficiently visible; distal radial physis visible if P-line assessment is planned.	Proceed to scenario-specific line or sign assessment.
No imaging evidence of radiocapitellar malalignment on adequate views	Projection adequate; line findings concordant across available projections; no ulnar bowing or plastic deformation; age-related eccentricity considered; clinical suspicion low or not discordant with imaging.	Structured report describing projection quality, line/sign used, ulnar morphology, limitations, and the need for clinical correlation if concern persists.
Equivocal	Projection limited; AP/lateral findings discordant; ossified landmarks insufficient; clinical findings discordant with apparent alignment; ulnar bowing present but line-based assessment uncertain.	Repeat standardized radiographs if feasible; otherwise use targeted problem-solving imaging based on the unresolved question.
Suspicious for Monteggia lesion	Clear malalignment on adequate imaging; malalignment with ulnar fracture or bowing; persistent abnormality after repeat standardized imaging; or high clinical concern with unresolved radiographic abnormality.	Consider orthopedic correlation/review and confirmation of alignment with repeat standardized imaging or targeted problem-solving imaging as clinically indicated.

**Table 7:** Everyday-use summary of the proposed interpretation framework. The table provides practical reporting support for common clinical imaging scenarios.

Scenario	First Question	Preferred Assessment	If Equivocal	Suggested Wording
Forearm trauma with ulnar fracture	Is the elbow included?	Dedicated elbow AP/lateral views; assess ulnar morphology.	Repeat elbow views if incomplete.	Radiocapitellar alignment cannot be confidently assessed on the current views.
Young child or low ossification	Are ossified landmarks reliable?	Lateral radial neck-axis RCL with ulnar morphology.	Consider US or MRI if findings remain discordant.	Alignment assessment is limited by incomplete ossification and should be correlated clinically.
Complete forearm radiograph with wrist	Are both radial physes visible?	P-line as a forearm-based adjunct.	Obtain dedicated elbow views if abnormal or unavailable.	P-line assessment does/does not suggest radiocapitellar malalignment on the available forearm views.
AP/coronal concern	Is lateral displacement suspected?	LHL as an AP/coronal adjunct.	Repeat standardized AP/lateral views or use problem-solving imaging.	AP adjunctive line findings are suspicious/limited; lateral-view correlation is required.
Ulnar bowing suspected	Is a true lateral forearm view available?	Ulnar bow sign/MUB.	Orthopedic review or targeted imaging if suspicion persists.	Ulnar plastic deformation raises concern for a Monteggia-equivalent injury.
Post-reduction or persistent concern	Is congruity or stability unresolved?	US, MRI, arthrography, or dynamic assessment according to the question.	Orthopedic correlation.	Targeted problem-solving imaging is suggested for the unresolved alignment question.

**Table 8:** Suggested structured reporting elements for suspected pediatric Monteggia lesions.

Reporting Element	What to Document	Example Wording
Radiographic adequacy	Whether elbow AP/lateral views are available, the lateral view is close to true lateral, and key landmarks are visible.	“Radiocapitellar assessment is limited because the lateral projection is not true lateral or ossified landmarks are insufficiently visible.”
Age/ossification context	Visibility of the capitellum and radial head, and whether age-related eccentricity may affect interpretation.	“RCL interpretation is limited by incomplete ossification; correlate with ulnar morphology and consider repeat or targeted problem-solving imaging.”
Line or sign used	RCL axis, P-line, LHL, radiocoronoid line, or ulnar bow sign/MUB.	“RCL was assessed using the radial neck axis on the lateral elbow view.”
Ulnar morphology	Ulnar fracture, bowing, or plastic deformation on a true lateral forearm view.	“Subtle dorsal ulnar bowing is present and should be interpreted with radiocapitellar alignment.”
Assessment category	No imaging evidence of radiocapitellar malalignment on adequate views, equivocal, or suspicious.	“Findings are equivocal because line findings and clinical concern are discordant.”
Suggested next step	Repeat radiographs, ultrasound, MRI, arthrography, or orthopedic review as clinically indicated.	“Repeat standardized views or targeted ultrasound/MRI may be considered for the unresolved alignment question. Persistent clinical concern may prompt orthopedic correlation.”

## Data Availability

No new clinical data were generated. The literature extraction table, search strategy, and main-text citation status of each record in the revised manuscript are provided as Supplementary Table S1. The main text cites the records most directly supporting the narrative and framework, whereas broader contextual records remain available in Supplementary Table S1.

## Supplementary Materials

The following supporting information can be downloaded at: https://www.mdpi.com/article/10.3390/diagnostics16132105/s1. Table S1: Evidence extraction table and search strategy for radiocapitellar alignment assessment in suspected pediatric Monteggia lesions. Table S1 includes the 57 unique literature records reviewed for this narrative synthesis, directness category definitions, Supplementary Methods S1 with the PubMed/MEDLINE search strategy, and each record’s main-text citation status in the revised manuscript. The main text cites the records most directly supporting the narrative and framework, whereas broader contextual records remain available in Table S1 and are included in the reference list here [25,42,43,44,45,46,47,48,49,50,51,52,53,54,55,56,57,58,59,60].

## Abbreviations

The following abbreviations are used in this manuscript:

AP	anteroposterior
LHL	lateral humeral line
MRI	magnetic resonance imaging
MUB	maximum ulnar bow
RCL	radiocapitellar line
US	ultrasound
